# Autism and Its Lookalikes: A Case Report of a Child Whose Autism Diagnosis No Longer Fit Years Later

**DOI:** 10.1155/crps/8279416

**Published:** 2026-02-09

**Authors:** Elizabeth Kim, Ji Su Hong

**Affiliations:** ^1^ Kennedy Krieger Institute, 1741 Ashland Avenue, Baltimore, 21205, Maryland, USA, kennedykrieger.org; ^2^ Department of Psychiatry and Behavioral Sciences, Johns Hopkin University School of Medicine, 733 N Broadway, Baltimore, 21205, Maryland, USA

**Keywords:** ADHD, autism spectrum disorder, co-occurring conditions, diagnostic stability, mood dysregulation

## Abstract

**Background:**

Autism spectrum disorders (ASDs) involve deficits in social communication and interactions as well as restricted, repetitive behaviors that can be reliably diagnosed in children as young as 14 months old, mostly by 36 months old, although signs of ASD may be present before then.

**Case Presentation:**

We present a case of a 5‐year‐old male who was diagnosed with ASD at 48 months and was found to no longer meet criteria for ASD upon reevaluation at 62 months after receiving medication treatment for his underlying attention‐deficit/hyperactivity disorder (ADHD) and severe mood dysregulation.

**Conclusions:**

In this report, we discussed the need to consider a broad differential of diagnoses that may resemble ASD and the need to reevaluate a child for ASD, especially if their ASD symptoms were mild on initial evaluation.

## 1. Introduction

Autism spectrum disorder (ASD) is defined by the Diagnostic and Statistical Manual of Mental Disorders‐Fifth Edition (DSM‐5) as having persistent deficits in social communication and interaction across multiple contexts as well as restricted, repetitive patterns of behavior, interests, or activities [1]. These symptoms must be present in the early developmental period, although they may not fully manifest until later in life where social and/or academic demands increase. Furthermore, the diagnosis is relatively stable over time [2]. The patient’s symptom severity is categorized into one of three levels: Level 1 meaning the patient requires support, Level 2 meaning they require substantial support, and Level 3 meaning they require very substantial support [1].

Salient to the case that follows, the Autism Diagnostic Observation Schedule, second edition (ADOS‐2), is often used as an observational assessment tool to diagnose ASD in people as young as 12 months old to adults and covers areas of communication, reciprocal social interactions, and restricted and repetitive behaviors associated with ASD [3]. It is considered to be the reference standard for evaluation of ASD with an interrater reliability of 0.79–0.98, sensitivity of 0.89–0.92, and specificity of 0.81–0.85 [4, 5].

Also pertinent to our case is the fact that people with ASD often lack theory of mind, which is the ability to understand other people’s mental states that may be different from one’s own [6]. The Sally and Anne test is a procedure that can be used to assess theory of mind [6]. In this test, the evaluator introduces Sally and Anne as two figures playing with a marble. Sally places the marble in a basket and leaves, after which Anne hides it in her box. Upon Sally’s return, the evaluator asks the patient where Sally would look for the marble. A child who has age‐appropriate theory of mind (around 60 months) would correctly answer “basket,” whereas a child who has not yet mastered age‐appropriate theory of mind would answer “box”.

Here, we present a case of a 5‐year‐old male who was previously diagnosed with attention‐deficit/hyperactivity disorder (ADHD) and ASD Level 1 at 48 months old, who came to a specialized ASD clinic 14 months later for diagnostic clarification and second opinion for his ASD diagnosis.

## 2. Case Presentation

The patient was a 5‐year and 2‐month‐old male who came to a specialized ASD clinic for diagnostic reevaluation. He had previously been diagnosed with ADHD and ASD Level 1 when he was 48 months old.

He has no history of seizures or head trauma. He had some growth restriction in utero but had no in utero exposure to substances or heavy metals. His birth was uncomplicated, and he did not require a NICU stay. He passed his hearing test at birth and at 3 years of age. His parents first became concerned about his development when he was 8 months old due to difficulty feeding in the setting of texture sensitivities, which improved with feeding treatment. At two and a half years of age, he began showing signs concerning ADHD. At 3 years old, he began exhibiting marked irritability, meltdowns lasting up to 2 h, and behavioral rigidity.

He had a psychological assessment at 48 months old. The Wechsler Preschool and Primary Scale of Intelligence‐4 (WPPSI‐4) was done, and his full‐scale intelligence quotient (FSIQ) was in the average range (Table 1). On his ADOS‐2 (Table 2), he scored 1 point for each item under the communication category: reporting events, conversation, and descriptive, conventional, instrumental, or informational gestures. Under reciprocal social interaction, he scored 1 point for quality of social overtures, quality of social response, and amount of reciprocal social communication. Under restrictive and repetitive behaviors, he scored a 1 on stereotyped/idiosyncratic use of words or phrases and on excessive interest in or references to unusual or highly specific topics or objects or repetitive behaviors. He scored 0 on all other items on the ADOS‐2 at the time. Overall, he scored 6 on the social affect and 2 on restricted and repetitive behaviors for a total score of 8. His score placed him in the autism spectrum classification and low level of autism spectrum‐related symptoms.

**Table 1:** Results of neuropsychiatric testing.

WPPSI‐4	FSIQ	Verbal comprehension	Visual spatial	Fluid reasoning	Working memory	Processing speed
SS	97	123	106	97	84	83

**Table 2:** ADOS‐2 scoring.

Age	48 months old	62 months old
Social affect (SA)		
(1) Communication		
A7. Reporting events	1	0
A8. Conversation	1	0
A9. Descriptive, conventional, instrumental, or informational gestures	1	0
(2) Reciprocal social interaction		
B1. Unusual eye contact	0	0
B2. Facial expressions directed to examiner	0	0
B4. Shared enjoyment in interaction	0	0
B7. Quality of social overtures	1	1
B9. Quality of social response	1	0
B10. Amount of reciprocal social communication	1	1
B11. Overall quality of rapport	0	0
Restricted and repetitive behaviors (RRBs)		
A4. Stereotyped/idiosyncratic use of words or phrases	1	0
D1. Unusual sensory interest in play material/person	0	0
D2. Hand and finger and other complex mannerism	0	0
D4. Excessive interest in or references to unusual or highly specific topics or objects or repetitive behaviors	1	1
Summary		
Social affect total (SA) score	6	2
Restricted and repetitive behavior total (RRB) score	2	1
Overall total (SA + RRB) score autism cutoff: 9 autism spectrum cutoff: 7	8	3
ADOS‐2 classification	Autism spectrum	Non‐spectrum
ADOS‐2 comparison score	5	1
Level of autism spectrum‐related symptoms	Low	Minimal‐to‐no evidence

He received applied behavioral analysis at 53–60 months old after he was diagnosed with ASD, which mainly focused on his outbursts and mood dysregulation rather than social communication skills or adaptive functioning, as the former were of utmost concern to his parents at the time. He also had treatment with medications since he was 53 months old. He had tried mixed amphetamine salts, both immediate‐ and extended‐release forms, although the extended‐release form was discontinued due to increased irritability. His most recent medication regimen included the immediate‐release form of mixed amphetamine salt 5 mg twice daily for ADHD, aripiprazole 2 mg twice daily for emotional dysregulation and aggression, and clonidine 0.1 mg every night for sleep. All three of those medications have been helpful for their respective purposes.

He underwent ADOS‐2 testing again at 62 months of age (Table 2). This time he scored a 1 for quality of social overtures and amount of reciprocal social communication under social affect, and he scored a 1 on excessive interest in or references to unusual or highly specific topics or objects or repetitive behaviors under restrictive and repetitive behaviors. He scored 0 on all other items, including the ones he previously scored a 1 on at 48 months, including reporting events, conversation, use of gestures, quality of social response, and stereotyped/idiosyncratic use of words or phrases. At 62 months, he scored 2 on social affect and 1 on restrictive and repetitive behaviors for a total score of 3 on the ADOS‐2. At that point, his score placed him in the non‐spectrum classification with minimal‐to‐no evidence of autism spectrum‐related symptoms.

In terms of his social development (Tables 3 and 4) [3], he met and demonstrated most of his social milestones on time including social smile, orienting to name, engaging in two‐way communication, playing peek‐a‐boo, waving, pointing, shaking/nodding his head, other conventional gestures, and descriptive gestures. He had some challenges with waiting his turn in playing games and engaging in cooperative play with peers before he was started on stimulant medications, although he has improved in both of these areas since starting medications.

**Table 3:** Social development expected by 12 months.

Social milestones in typical development	Around 12 months old
Social smiles (8 weeks. By 3 months)	Yes
Orients to name well (9 months. By 12 months)	Yes
Engage in back‐and‐forth, two‐way communication using vocalization and eye contact (by 9 months)	Yes
Play peek‐a‐boo (10 months. By 12 months)	Yes
Waves bye‐bye (10 months. By 12 months‐imitation. By 15 months‐use)	Yes
Proto‐imperative pointing to get desired objects (by 12 months)	Yes

**Table 4:** Progress of social development with medications.

Social milestones in typical development	Current social function with medications
Social reciprocity and relationship	
Reciprocal smiling (2 months. By 3 months)	Yes
To and fro alternating vocalizations (4 months) Engage in back‐and‐forth, two‐way communication using vocalization and eye contact (by 9 months)	He makes good turn‐taking conversation when the topic is of his interest. If the topic is not interesting, he would ignore or interrupt to bring his topics.
Enjoy extended play with others, especially care‐givers (by 9 months)	Yes. Observed at the session.
Seeks and enjoys attention from others, especially caregivers (by 15 months)	Yes
Can wait turn in playing games (by 36 months)	It has been better with medications.
Starts to share with/without prompt (3 years)	Yes
Engages in cooperative play with other infants/young children (by 48 months)	Better with medications
Labels happiness, sadness, fear, and anger in self Uses “feeling” words (4 years)	Yes
Passes Sally and Anne test (4 years)	He failed at the session.
Modulates or modifies voice correctly depending on situation or listener (e.g., outside voice, to adult, other infant/young child, or younger child) (by 60 months)	Yes. Observed at the session.
Looks from object to parent and back when wanting help (initiation of joint attention, 7 months, by 9 months)	Yes. Observed at the session.
Engages in gaze monitoring: adult looks away and child follows adult glance with own eyes (8 months)	Yes Observed at the session.
Follows a point, “Oh look at…” (by 9 months)	Yes. Observed at the session.
Orients to name well (9 months. By 12 months)	Most time
Give to initiate interaction or get help (9 months. By 12 months)	Yes. Observed at the session.
Shows objects to parent to share interest (by 12 months)	Yes. Observed at the session.
Nonverbal communication	
Eye‐contact	Good
Proto‐imperative pointing to get desired objects (by 12 months)	Yes
Proto‐declarative pointing to express/share interest (by 15 months)	Yes. Observed at the session.
Uses complex communication skills integrating gestures, vocalizations, and eye contact (e.g., looking to parent while taking his or her hand to bring him or her to a desired toy. By 15 months)	Yes. Observed at the session.
Shakes head for no (8 months. By 15 months)	Yes. Observed at the session.
Nodding head for yes (15 months)	Yes
Waves bye‐bye (10 months. By 12 months‐imitation. By 15 months‐use)	Yes
Clap (13 months)	Yes
Blow a kiss (13 months)	Yes
Shh gesture (14 months)	Yes
Thumbs up (15 months)	Yes
High five (16 months)	Yes
Conventional gestures	Observed at the session.
Descriptive gesture	Observed at the session.

One notable finding from our evaluation was that he had failed the Sally and Anne test, indicating difficulty with theory of mind as expected for his age. We concluded that our patient did not have ASD, although failing the Sally and Anne test was concerning and warranted further monitoring.

He was diagnosed with ADHD combined presentation and other specified disruptive, impulse‐control, and conduct disorder. The patient’s family was advised to continue monitoring him and to consider reevaluation in another year if his symptoms worsen or if new concerns arise.

## 3. Discussion

Our patient’s developmental history is not consistent with ASD. Per the diagnostic criteria of ASD, one must have persistent deficits in social communication and social interactions across multiple contexts to be diagnosed with ASD [1]. Congruent with this diagnostic criteria, a systematic review by Woolfenden et al. [2] showed that ASD is a relatively stable diagnosis over time. However, our patient’s deficits in social communication and interactions were not consistent over time and with medications.

Once his ADHD and severe mood dysregulation were treated with medications, his ADOS‐2 score dropped to the non‐spectrum range with minimal‐to‐no‐evidence of ASD‐related symptoms. The change in his ADOS‐2 score was not explained by ASD but rather by the management of his ADHD and mood dysregulation, which resembled ASD when he was younger. It is worth noting that while aripiprazole has been shown to improve irritability and aggression associated with ASD [7–11], it would not necessarily improve the core features of ASD, particularly social communication and reciprocal interactions, which changed over time for our patient.

Our patient demonstrated most of his social milestones except that he failed the Sally and Anne test, which typically developing children are usually expected to pass by age 4 and age 5 at the latest [12]. It should be noted that the specificity of this test for diagnosing ASD is controversial, as theory of mind deficits are not limited to children with ASD [13]. While many children with ASD lack theory of mind due to their deficits in social interactions [12], theory of mind can be a function of one’s language abilities since language is known to be a predictor of communication and socialization [14]. For this reason, social pragmatic communication disorder remained on the differential for consideration. Theory of mind can also be influenced by executive functioning skills since working memory and inhibitory control also account for one’s ability to reflect on other people’s mental states and to show their understanding to the examiner [15]. In this sense, our patient’s difficulty with theory of mind in the Sally and Anne test at the time may also be a reflection of his underlying ADHD.

It is also possible that other factors contributed to our patient’s change in presentation over time. Increased opportunities for socialization as he started kindergarten during the period between his ADOS‐2 measurements may have helped diminish his score on ADOS‐2, especially in the social communication scores. Although ABA focused mostly on outbursts for our patient rather than social skills or adaptive functioning, it may have also had a positive impact on his social communication and restrictive, repetitive behaviors, as behavioral therapies have been shown to be effective in addressing those features [10, 11]. He was also taking clonidine for sleep, which may have had a positive impact on his ADOS‐2 score given that inadequate sleep has been linked with difficulties in socioemotional and cognitive functioning [16, 17].

The diagnosis of ASD should be made with careful consideration in the context of its clinical and administrative consequences. Having the diagnosis opens doors for early interventions tailored to the specific needs of the child, individualized education plans that help the child maximize their classroom education, as well as home and community resources, along with financial assistance for services that can improve the quality of lives for children and their families [18, 19]. Diagnosing a child with ASD also comes with the risk of stigmatization, which can negatively impact the child’s self‐attitude and social interactions, and the risk of overdiagnosing or misdiagnosing the child, which can result in unnecessary treatments. Furthermore, an ASD diagnosis also comes with the risk of diagnostic overshadowing and delaying the identification and treatment of other disorders such as ADHD, and anxiety [18]. Thus, an ASD diagnosis is not one to be made lightly, especially in the setting of symptoms that overlap with other disorders.

## 4. Conclusions

As shown by our case example, one’s ADOS‐2 score can be fluid rather than fixed and may warrant reevaluation in the future, especially if the patient scored low in the autism spectrum range, as our patient did. If the score changes over time, one should think about what other diagnoses can explain the patient’s presentation. If the patient scores 0’s and 1’s on the ADOS‐2, one should suggest follow‐up as the patient is treated for any co‐occurring disorder they might have. One should keep in mind that diverse developmental and mental disorders can resemble ASD, which necessitates the need to treat underlying conditions and reassessing, especially if the patient has mild ASD‐like symptoms. It would be important to explain to the caregiver the overlap of symptoms across various disorders, the meaning of their child’s score on ADOS‐2, and the rationale for follow‐up and reassessment.

## Funding

No funding was received for this manuscript.

## Conflicts of Interest

The authors declare no conflicts of interest.

## Data Availability

All data supporting the findings of this case report are available within this paper.
